# Biliary Hyperkinesia and Its Association With Bariatric Surgery: A Case Report and Review of Management Strategies

**DOI:** 10.7759/cureus.34119

**Published:** 2023-01-23

**Authors:** Shaniah S Holder, Ulochi Nwagwu, Farhana Ghouse, Muhammad Zain Ali, Frederick Tiesenga

**Affiliations:** 1 Department of Medicine, American University of Barbados School of Medicine, Bridgetown, BRB; 2 Department of Surgery, West Suburban Medical Center, Chicago, USA; 3 Department of Medicine, St. George's University School of Medicine, True Blue, GRD; 4 Department of Medicine, Saint James School of Medicine, Park Ridge, USA; 5 Department of General Surgery, West Suburban Medical Center, Chicago, USA

**Keywords:** cholecystectomy, ejection fraction, hida scan, biliary dyskinesia, functional gallbladder disorder, sleeve gastrectomy, hyperactive gallbladder, bariatric surgery

## Abstract

In a post-bariatric surgery patient with suspected biliary dyskinesia, what does an ejection fraction (EF) of 87% on hepatobiliary iminodiacetic acid (HIDA) scan indicate to a healthcare provider? Conventionally, in post-bariatric patients, the gallbladder becomes hypofunctional; however, in this case, the gallbladder activity increased exponentially. Of note, there are no previously documented cases of developing an overactive gallbladder after undergoing a bariatric surgery procedure. This report aims to explore the possible associations between bariatric surgery and the development of gallbladder hyperkinesis in the early postoperative period, the diagnostic tool used to discover the source of our patient’s ailment, as well as the rationality behind a surgical procedure that led to an excellent response, namely, laparoscopic cholecystectomy.

## Introduction

Obesity is the number one risk factor for developing gallbladder disease. The incidence of such illnesses is directly correlated to the body mass index (BMI) [1]. However, rapid weight loss increases the mobilization of cholesterol, resulting in an increased biliary concentration and subsequently the development of cholelithiasis [1]. In treating severe obesity, bariatric surgery has been beneficial in improving or resolving metabolic comorbidities such as type II diabetes mellitus and increasing life expectancy [2]. Using either a restrictive, malabsorptive, or a combination of the two processes, bariatric surgery involves the manipulation of the gastrointestinal (GI) tract to achieve quantifiable weight loss [1]. These procedures include gastric banding, sleeve gastrectomy (SG), biliopancreatic diversion, duodenal switch, and Roux-en-Y gastric bypass (RYGB) [1,3]. Bariatric surgery does not only affect the stomach and intestines, the gallbladder is inadvertently affected as well.

The incidence of gallbladder disease post-bariatric surgery is maximal during the period of rapid weight loss which occurs within the first six months, regardless of the type of surgery [4]. Studies have shown this risk decreases after weight stabilizes [4]. The formation of gallstones due to rapid weight loss involves cholesterol supersaturation of bile, decreased secretion of biliary acids due to caloric restrictions, increased mucin production which enhances crystallization, and finally, gallbladder hypomotility secondary to decreased cholecystokinin secretion related to the hypocaloric diet [2,4].

In this case, we use cholecystokinin 99m technetium-labeled hepatoiminodiacetic acid (99mTc-HIDA (CCK-HIDA)) for the patient’s workup as studies have shown it is the most accurate modality for diagnosing gallbladder disease by determining gallbladder contractility and ejection fraction (EF) [5]. Gallbladder hypomotility also known as biliary dyskinesia is a common functional biliary disorder defined by an EF of less than 35% on a 99mTc-HIDA (CCK-HIDA) scan [6]. It is standard to perform a cholecystectomy in the management of this condition with effective results. However, there is a paucity of research and management of patients with biliary colic symptoms and high gallbladder EF [6]. A gallbladder EF of 80% or greater is commonly used to define biliary hyperkinesis also known as hyperkinetic gallbladder [6]. The hyperkinetic gallbladder is characterized by rapid contractions and emptying of the gallbladder. Herein, we present a patient with symptomatic biliary hyperkinesis one-month post-bariatric SG, who had an excellent response to laparoscopic cholecystectomy.

## Case presentation

The patient, in this case, is a 37-year-old female with a past medical history of morbid obesity (BMI 47 kg/m^2^) who presented to the emergency department with nausea, vomiting, right upper quadrant (RUQ) pain, fatigue, and constipation. One month prior to her presentation, she underwent a laparoscopic SG with no surgical complications and a normal post-op period and was subsequently discharged. She stated that she had been unable to tolerate most foods and liquids since two weeks post-op. It was noted that the patient lost 11.3 kg (BMI 44.3 kg/m^2^) within the one month following the procedure.

Upon examination, the patient had upper quadrant tenderness that was worse on the right and exacerbation of pain and nausea on palpation; thus, further workup was performed. The patient’s past surgical history of SG indicated laboratory tests with a focus on the patient’s liver function tests (LFTs), in which the results were unremarkable.

There was no evidence of gallbladder wall thickening, pericholecystic fluid or gallstones visualized on computed tomography (CT) of the abdomen and pelvis with intravenous (IV) contrast. Additionally, an esophagogastroduodenoscopy (EGD) and endoscopic ultrasound (EUS) were performed to exclude gastritis, stricture, gastric twist, peptic ulcer disease (PUD), and gallstones. These reports also demonstrated unremarkable results; with no stones or bile duct dilation visualized and benign findings of chronic inactive gastritis. The Gastroenterology department declined to conduct a magnetic resonance cholangiopancreatography (MRCP) because the patient did not meet the criteria of elevated LFTs and dilated common bile duct.

Due to the normal labs and imaging, gallbladder pathology was suspected to be the source of her symptoms; therefore, a functional HIDA scan with CCK administration was conducted. The bile ducts were clearly visualized and patent; however, an atypical finding was the gallbladder EF, which was noted to be 87%, indicating a hyperactive gallbladder, instead of the conventional sluggish activity normally noted after bariatric surgery. The patient reported that she experienced nausea and abdominal pain after CCK was administered.

**Figure 1:**
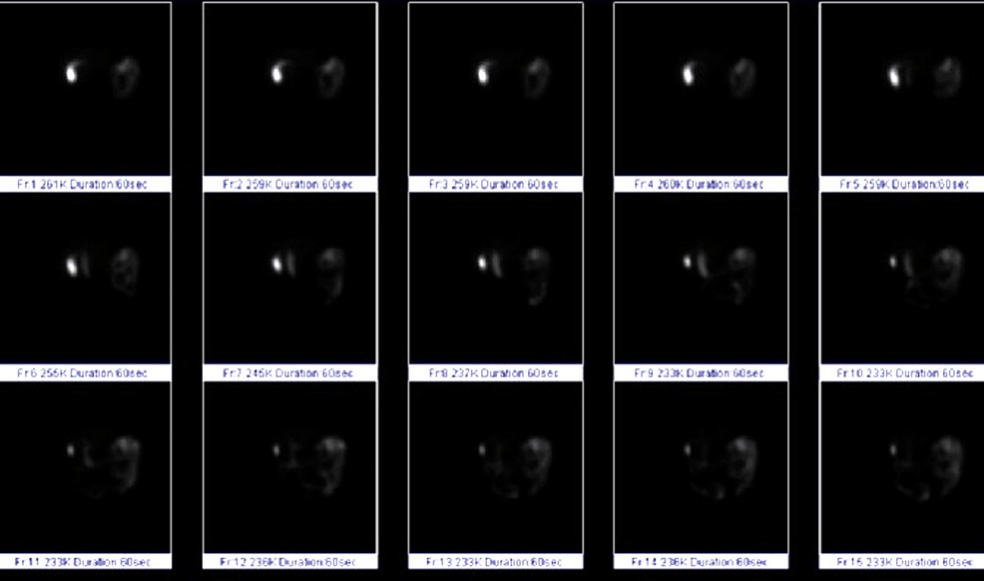
Summary of HIDA scan findings showing 87% of contrast leaving the gallbladder during contraction. HIDA: hepatobiliary iminodiacetic acid scan

Figure 1 below summarizes the HIDA scan findings which showed contrast in the gallbladder (row 1, image 1) followed by contraction and subsequent ejection of 87% of contrast with less than 13% remaining in the gallbladder after 60 minutes (row 3, image 5).

Due to positive diagnostic findings on the HIDA scan, there was a high clinical suspicion that her hyperkinetic gallbladder was the cause of her symptoms; therefore, ROME IV criteria for the functional biliary disorder was reviewed. Table 1 below shows the symptomatic criteria required for diagnosis. In this case, the patient’s symptoms fit the ROME criteria with the exception of low EF on gallbladder scintigraphy, which in her case was elevated.

**Table 1:** ROME IV criteria to evaluate for functional gallbladder disorder. [7] * criteria that support gallbladder dysfunction, but are not required for diagnosis HIDA: hepatobiliary iminodiacetic acid

ROME IV Criteria for Functional Gallbladder Disorder
Biliary pain
Absence of gallstones or other structural pathology
Pain located in the right upper quadrant or epigastrium
Pain builds to a steady level and lasts at least 30 minutes
Occurs at variable intervals, not daily
Not significantly (< 20%) relieved by postural change, acid suppression, or bowel movement
Is severe enough to disrupt the daily activity or lead to an emergency department visit
Low ejection fraction on HIDA scan*
Normal liver enzymes, conjugated bilirubin, amylase, and lipase*

**Figure 2:**
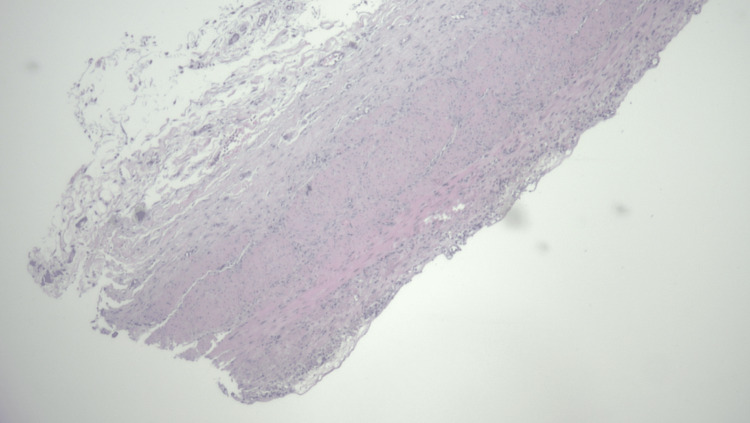
Microscopic view of the thickened gallbladder wall.

The patient was informed of her diagnosis of the hyperkinetic gallbladder and that this condition was possibly responsible for her symptoms. She was also notified that the removal of her gallbladder may not relieve her symptoms and with her understanding, an elective laparoscopic cholecystectomy was performed. The procedure was well tolerated and without complication. The gallbladder was 3.8 x 2 x 2 cm in size. Postoperative gallbladder pathology reported incidental small green-black stones with the largest stone being 0.8 cm in diameter. Gallbladder wall thickness was noted to be 0.3 cm indicating mild chronic cholecystitis. Figure 2 shows gallbladder smooth muscle hypertrophy due to excessive contraction.

Postoperatively, the patient stated that her symptoms were completely resolved. A clear liquid diet was started on postoperative day one, which was well tolerated with no further report of nausea or pain. She was instructed to advance her diet as tolerated and was discharged with a plan to follow up.

## Discussion

The etiology of gallbladder hyperkinesis and its association with bariatric surgery is unclear due to the scarcity of research on this topic; however, some literature suggests that abnormal gastric emptying, changes in bile composition, and the hormone CCK play a role [8].

Bariatric surgery is a group of exceptional weight loss procedures that helps millions of people per year. It alters GI anatomy and physiology leading to changes in the volume of secretions and actions of gut hormones [9]. Among the many common complications of bariatric surgery, increased gastric motility manipulates GI hormone production [10]. The hormone of focus in this study is CCK, which is secreted by duodenal I cells in response to gastric emptying and leads to gallbladder contraction and relaxation of the sphincter of Oddi [5]. However, this hormone also delays gastric emptying in persons with increased gastric motility. It acts as negative feedback by causing contraction of the pyloric sphincter and relaxation of the proximal stomach [11]. Studies show that increased postprandial CCK inhibits gastric emptying of liquids and semisolid meals and hormone concentrations have been reported to increase after bariatric surgery, especially post RYGB and SG [12]. Increased CCK levels or increased gallbladder sensitivity can lead to hyperkinesis of the gallbladder and must be viewed as a possible underlying etiology of the hyperkinetic gallbladder in post-bariatric patients [13].

Biliary hyperkinesis and dyskinesia present with similar symptoms with the only exception being EF [14]. Increased force of contraction leads to an elevation in gallbladder pressure which manifests as biliary colic characterized by RUQ pain with radiation to the right shoulder, nausea, vomiting, diaphoresis, and general weakness [8]. Symptoms occur at variable intervals, not daily, and may not be associated with food intake. Biliary hyperkinesia is a diagnosis of exclusion. LFTs (aspartate aminotransferase (AST), alanine aminotransferase (ALT), alkaline phosphatase (ALP), bilirubin), amylase, and lipase levels as well as imaging with U/S or CT are the initial steps of diagnosis and are usually normal with minimal-to-no evidence of gallstones, sludge, or cholesterol polyps in patients with hyperkinetic gallbladder [15]. In patients with normal U/S, upper endoscopic tests (EUS, EGD) can be performed to rule out PUD or microlithiasis. In the absence of other GI causes of biliary pain, a HIDA scan with CCK also known as CCK-stimulated cholescintigraphy can be used to evaluate the EF. A 99mTC-HIDA scan is an imaging procedure that is used to visualize the function of the hepatobiliary system, especially the gallbladder. During a HIDA scan, radioactive tracer and CCK are injected into a vein in the arm, the contrast travels through the liver, gallbladder, bile ducts, and small intestines and the CCK stimulates gallbladder contraction. A camera is then used to take a series of images of the tracer as it moves along [16]. HIDA scans have excellent sensitivity and accuracy in the diagnosis of functional gallbladder disorders [6]. An EF reading of >80% is specific for gallbladder hyperkinesis. CCK infusion may lead to the reproduction of biliary symptoms in some cases, however, this is not specific [14]. Chronic cholecystitis is a common sequela in persons with biliary hyperkinesia [17]. Increased gallbladder emptying is associated with increased intraluminal pressure which causes mucosal injury and inflammation and leads to gallbladder wall thickening [13]. The pathology report of our post-bariatric patient’s gallbladder showed mild chronic cholecystitis. Although postoperative incidental small stones were found, this was noncontributory based on the stone size, patency of the biliary ducts, both cystic and common, and an elevated EF of 87%; indicating that biliary blockage with gallstones was not the cause.

Management of gallbladder hyperkinesis can be categorized into nonsurgical and surgical methods. Diet modification, antiemetics, and analgesics are used for symptomatic management. Surgical treatment via laparoscopic cholecystectomy is noted to be the gold standard therapy for gallbladder hyperkinesis and is superior in symptom resolution when compared to nonsurgical modes of management [18]. In our case, the patient reported a complete resolution of symptoms within two days post-cholecystectomy. It is inferred that persons who take a surgical approach tend to have a lower incidence of emergency department visits and an improved quality of life in comparison to their non-surgical counterparts [18].

Gallbladder hyperkinesis could be one of the rarer complications of the early post-op period of bariatric surgery and further research into this association is required. Physicians should be aware of this unusual relationship and respond appropriately by performing diagnostic tests such as HIDA scans to establish a definitive diagnosis. Biliary hyperkinesis, following bariatric surgery or not, should be considered in a patient with an EF of more than 80%. The presence of biliary symptoms and reproducibility of symptoms, when exposed to CCK, should indicate cholecystectomy since these findings suggest increased response to surgical treatment.

## Conclusions

An uncommon occurrence termed hyperkinetic gallbladder, defined by an EF of greater than 80% on a HIDA scan, may be observed in post-bariatric surgical patients. There are no previously documented cases of developing an overactive gallbladder after undergoing bariatric surgery. Our case presents an unexplored post-operative complication and further demonstrates the effectiveness of performing a cholecystectomy on symptomatic hyperkinetic gallbladder disease.

The patient involved in this case study endorsed unrelenting nausea, vomiting, anorexia, and abdominal pain, which was also reproduced when exposed to CCK. These aforementioned symptoms were coupled with vague laboratory values and unremarkable diagnostic imaging via CT scan and EUS, particularly the absence of gallbladder wall thickening, pericholecystic fluid, or gallstone visualization. A remarkably high EF of 87% indicated by the HIDA scan changed the course of the diagnosis and led to a potential management route for the patient’s distress. This was effectively demonstrated by the complete resolution of symptoms upon receiving a cholecystectomy; thus, it should be strongly considered as a treatment option by healthcare providers. Overall, there is an important correlation between biliary hyperkinesia and bariatric surgery which requires further exploration, particularly, the use of HIDA scans to establish a definitive diagnosis.
